# Supplementary material for: Validation of web-based audiometry version of HEARZAP
Source: PLoS One. 2023 Mar 28;18(3):e0283519. doi: 10.1371/journal.pone.0283519 (PMC10047537; doi:10.1371/journal.pone.0283519)
Supplement: Attachment — Submitted filename: Response to Reviewers - PLOS.docx [file pone.0283519.s001.docx]

21^st^ February 2023

To

The Editor

PLOS ONE

Dear Editor,

We thank the reviewers for providing comments on the manuscript titled “**Validation of web-based audiometry version of HEARZAP**” for consideration in PLOS ONE. We have addressed the journal requirements and Reviewers' comments below. We have no restrictions in sharing the data set. We have uploaded the data set in the Open Science Framework repository and it can be accessed through the link <http://osf.io/x3svw.>

Truly,

Vidya Ramkumar

**Reviewers' comments:**

1. **Criticism: - Lacking word and error in abstract;**

Author’s Response: We have corrected the word and grammatical errors in abstract.

2. **Please write “Figure 1” as “Fig 1” and so on;**

Author’s response: “Figure 1” has been changed to “Fig 1” and so on throughout the manuscript.

3. **Please insert Fig 1 citation in the text;**

Author’s response: Fig 1 citation has been included in the manuscript on page no. 9 and 10.

4. **Please correct extra space in the text;**

Author’s response: Extra spaces have been corrected in the manuscript. However, when document is viewed in different PC’s or Mac, spacing between words seems to change. We hope this can be taken care during final editing.

5. **Please cite references in brackets in the text.**

Author’s response: References have been cited in brackets according to PLOS One guidelines.

**Journal Requirements:**

1. Please ensure that your manuscript meets PLOS ONE's style requirements, including those for file naming.

**The submitted manuscript meets PLOS ONE's style requirements, including those for file naming as per guidelines.**

2. Please include your full ethics statement in the ‘Methods’ section of your manuscript file. In your statement, please include the full name of the IRB or ethics committee who approved or waived your study, as well as whether or not you obtained informed written or verbal consent. If consent was waived for your study, please include this information in your statement as well.

**Full ethics statement has been updated in ‘Methods’ section of the manuscript in page no.7.**

3. We note that you have indicated that data from this study are available upon request. PLOS only allows data to be available upon request if there are legal or ethical restrictions on sharing data publicly. For more information on unacceptable data access restrictions, please see http://journals.plos.org/plosone/s/data-availability#loc-unacceptable-data-access-restrictions.

**We have no restrictions in sharing the data set. We have uploaded the data set in the Open Science Framework repository and it can be accessed through the link** [**http://osf.io/x3svw.**](http://osf.io/x3svw.)

4. Please review your reference list to ensure that it is complete and correct. If you have cited papers that have been retracted, please include the rationale for doing so in the manuscript text, or remove these references and replace them with relevant current references. Any changes to the reference list should be mentioned in the rebuttal letter that accompanies your revised manuscript. If you need to cite a retracted article, indicate the article’s retracted status in the References list and also include a citation and full reference for the retraction notice.

**The reference list has been checked and it is complete.**
